# Assessment of health services for people who use drugs in Central Asia: findings of a quantitative survey in Kazakhstan and Kyrgyzstan

**DOI:** 10.1186/s12954-016-0093-2

**Published:** 2016-01-27

**Authors:** Moritz Rosenkranz, Nina Kerimi, Madina Takenova, Antti Impinen, Mirlan Mamyrov, Peter Degkwitz, Heike Zurhold, Marcus-Sebastian Martens

**Affiliations:** Department of Psychiatry and Psychotherapy, Center for Interdisciplinary Addiction Research of Hamburg University (CIAR), University Medical Center Hamburg-Eppendorf, Martinistraße 52, 20246 Hamburg, Germany; Regional Office for Central Asia, United Nations Office on Drugs and Crime (UNODC), 26 Bukeykhan Street, UN House, 010000 Astana, Kazakhstan

**Keywords:** PWUD, Central Asia, Kazakhstan, Kyrgyzstan, Opioid drug use, Access to health care, Harm reduction, Barriers to service utilization, HIV, HCV

## Abstract

**Background:**

In Central Asia, there is a need to update information about the situation of people who use (opioid) drugs (PWUD), especially regarding their access to and utilization of health care services. The aim of the study was to gather information about two different groups of drug users in Kazakhstan and Kyrgyzstan.

**Methods:**

In 2013, two groups of PWUD were recruited in Kazakhstan and in Kyrgyzstan in order to gather quantitative data via interviewer-administered questionnaires. PWUD registered with the Narcological Register were allocated to group A while non-registered PWUD were allocated to group B. Interviews were conducted in the office of the Narcological Register as well as in low-threshold facilities. Participants reported about their drug use patterns, health status, and utilization of health services as well as barriers to utilization.

**Results:**

The sample consisted of *N* = 600 PWUD (301 registered and 299 non-registered PWUD) from Kazakhstan and *N* = 900 PWUD (450 registered and 450 non-registered PWUD) from Kyrgyzstan. Both groups—registered (group A) and non-registered (group B)—consisted of mainly male long-term intravenous opioid users. We found high rates of current (last 30 days) opioid use (group A up to 70 %; group B up to 84 %). Most PWUD were burdened with poor physical and mental health. The prevalence of infectious diseases added up to 19 % (group A) or 13 % (group B) regarding HIV, 56 % (group A) or 30 % (group B) regarding HCV, and 24 % (group A) or 20 % (group B) regarding tuberculosis. Registered and non-registered PWUD reported high rates (95 or 82 %) of lifetime use of health services for PWUD. Drug-related services were utilized less often, especially among the non-registered PWUD (13 %). The most important barriers preventing PWUD from accessing services were the belief not to need treatment, doubts about the effectiveness of treatment, mistrust of treatment regime/staff, and fear of being registered with the Narcological Register (mainly group B).

**Conclusions:**

Results show that access to the health care system for non-registered PWUD is realized mainly through low-threshold facilities. Opioid substitution treatment, which is an important pillar in the treatment of PWUD, is normally only available for those registered with the Narcological Register. Instead, access to opioid substitution treatment (especially in Kazakhstan) should be expanded and granted without prior registration, as this poses an important barrier for PWUD’s utilization of drug treatment services. Further, there seems to be a need for the provision of specific and target group-related information about drug treatment services in order to reduce existing reservations among PWUD as to the necessity and effectiveness of modern drug treatment.

## Background

Kazakhstan and Kyrgyzstan are two post-Soviet republics located in Central Asia. Kazakhstan’s territory adds up to 2,724,900 km^2^. Even though the country is larger than Western Europe, only approximately 17.5 million people (of mostly Kazakh and Russian ethnicity) live in Kazakhstan [1]. The World Bank classified Kazakhstan as an “upper-middle-income country with per capita GDP of nearly US$13,000 in 2013.” [2].

Kyrgyzstan, extending to 198,500 km^2^, has a population of 5.5 million people [3]. The biggest ethnicities are Kyrgyz, Uzbek, and Russian. In comparison with Kazakhstan, Kyrgyzstan is a much poorer country (per capita GDP US$1300) [4].

The development of the health care system in general and of specific drug treatment programs in particular is of well-known significance to reducing the public health-related consequences of substance use, especially the incidence of HIV among people who use drugs (PWUD)^1^ [5]. This interrelation is of particular relevance to young post-Soviet countries in Central Asia such as Kazakhstan and Kyrgyzstan for two reasons: Firstly, the World Drug Report 2015 [6] has indicated one of the highest annual prevalence rates for the opioid use in the world for this region (0.9 %). Secondly, HIV prevalence among PWUD in Central Asia is high and still rising [7–9] (see below).

In the past few years, many international and Central Asian stakeholders have repeatedly pointed out the need to update the drug use-related data for the region of Central Asia: “At present it is extremely difficult to obtain an objective picture of drug consumption both among the general population and among specific groups […] because of the lack of a good-quality epidemiological study in the country.” [10]. Though there are miscellaneous reports related to drug use, drug trafficking, and drug seizures [11, 12], “reliable data on drug use and its patterns in the general population of the countries of Central Asia are not available” [13]. Some information about the drug situation in Central Asia is only based on experts’ opinions; the last survey on the drug situation in Kazakhstan was conducted in 2001 and suffers from methodological deficits (regarding sampling, questionnaire, etc.) which make it difficult to generalize its results [10, 13]. This study reported that 1.7 % of the general population used drugs. Among these, 31.6 % were dependent on opioids and 81.3 % on cannabis [14]. The estimate of problem drug use (injecting drug use) in the previous 12 months among the general Kazakh population in 2012 was 1 % [15].

Regarding Kyrgyzstan, drug use among the general population was examined in an estimation study in 2002, but detailed information on the methodology of the study is lacking. The authors reported 2.6 to 3.3 % of the population (16 to 64 years old) as drug users and 1.8 % of the population as problem drug users [16].

For some countries—Kazakhstan and Kyrgyzstan, for example—government statistics can be found: “Narcological centers” (NCs), which are located in all provinces, are in charge of PWUD and their treatment. PWUD who access the public drug treatment system or come to the attention of the police for an offense have to undergo a urine test for illegal drugs. If the result of the test is positive, they will be registered. On first contact to the NC, a person is registered either in the “dispensary narcological register” (if diagnosed according to ICD-10) or in the “prophylactic narcological register” (if no addiction was diagnosed). NCs collect sociodemographic data as well as drug use data (incl. possible diagnoses) and the results of somatic and toxicological medical examinations of PWUD in a database called the “Narcological Register.” As law enforcement agencies have access to personal data in the register, the Narcological Register is also used, e.g., to prevent PWUD from driving, possessing a weapon, or to ban them from different fields of employment, such as the military, the police, or the educational system. Once registered, the PWUD has to appear in person at the NC for a quarterly examination by a narcologist (specialized medical doctor). In order to be removed from the Kazakh “dispensary register,” the PWUD have to show abstinence from illegal drugs and alcohol for at least 5 years; in Kyrgyzstan, this is the case after 3 years. PWUD who are in the “prophylactic register” will be removed if they prove to be abstinent for 3 years (in Kazakhstan) or 1 year (in Kyrgyzstan). Further reasons for deletion from the register are a move to a region outside the territory covered by the NC, imprisonment, or death [10, 17]. In 2011, 30,259 PWUD were registered with narcology in Kazakhstan [10]. Regarding Kyrgyzstan, the only data available are from 2008: 9057 PWUD were registered with narcology in this country [18].

Drug treatment in Kazakhstan is free of charge (except for drug treatment offered by the very confined private narcological sector) [10]; in Kyrgyzstan, the expenses are covered by the patients or the medical insurance [18]. In 2013, a group of treatment experts were asked to assess the availability of key treatment offers in different Central Asian countries: They reported detoxification to be fully available in Kazakhstan as well as in Kyrgyzstan, whereas the availability of inpatient drug-free medical treatment was described as moderate in both countries. Outpatient drug-free medical treatment was fully available in Kazakhstan; in Kyrgyzstan, only moderately. Availability of opioid substitution treatment (OST) was assessed as rare regarding Kazakhstan and as moderate regarding Kyrgyzstan. Psychological support and therapy was available moderately in both countries, whereas social rehabilitation was available only rarely in Kazakhstan and Kyrgyzstan [13].

In 2011, 2972 PWUD underwent treatment in Kazakhstan, and 3277 PWUD in Kyrgyzstan [10, 13]. Treatment rates (PWUD treated per estimated number of all PWUD) are significantly higher in Kyrgyzstan (17.3 %) than in Kazakhstan (2.5 %). In 2011, OST was provided by three units in Kazakhstan, serving 115 PWUD (detoxification, 33 units; 1579 patients). In Kyrgyzstan, 20 units provided OST in 2011, covering 1428 PWUD (detoxification, 50 units; the exact number of patients undergoing detoxification was indeterminate) [10, 13, 16]. Eligibility criteria for OST in the region are not clearly defined. Among others, the main criterion is a history of unsuccessful narcological treatment attempts. Occasionally, there are special “commissions” (sometimes they include members without any medical background) that decide about each single PWUD’s access to OST [19]. The number of injecting drug users who had contact to harm reduction programs in 2011 in Kazakhstan was 79,579; in Kyrgyzstan, the respective number is only available for 2010 and amounts to 9120 persons [13].

Data from the Narcological Register only reflect a small part of the actual situation as they only cover approximately one third (44,825 persons) of the estimated injecting PWUD (123,640 persons) in Kazakhstan [10]. A lack of information exists especially concerning the situation and the utilization of services of PWUD who are not registered with the NCs. Another data source is the Sentinel Epidemiological Surveillance which is conducted under the supervision of the “Republican AIDS Centre” [15]. These investigations are mainly concerned with gaining epidemiological information in Kazakhstan and therefore only partly cover the health service situation regarding PWUD. Additionally, the quality of these data and information is weak due to methodological problems. The methodology of the sampling procedure as well as the calculation of the sample size is assessed as incorrect; interviewing techniques, questionnaire design, and data analysis, e.g., generalization of the results on a national level, are considered as questionable [10].

As illustrated, there is a need for updated data on the (health service) situation of PWUD in Central Asia. Furthermore, the incidence of HIV among PWUD is high (KAZ 2011, 3.8 %; KRG 2010, 14.6 %) [10, 16] and—in most of the region’s countries—continues to increase [7, 8] in spite of many efforts by national governments, NGOs, and international development aid organizations. The proportion of PWUD infected with HCV among all PWUD is reported as 61.3 % for Kazakhstan (2011) and as 50.4 % for Kyrgyzstan (2010) [10, 16]. These facts led the UNODC Regional Office for Central Asia to initiate a comprehensive study titled: “Assessment of adequacy of health services for people who use drugs (PWUD) in countries of Central Asia and in the Republic of Azerbaijan.” The study protocol along with the study instruments (questionnaires, templates, guides, and forms) was developed by members of the Centre for Interdisciplinary Addiction Research of the University of Hamburg (CIAR) in close cooperation with the UNODC project staff. Selected results from this study are presented in this article.

The study aimed not only to facilitate a better understanding of the drug-using population but also to allow for an appraisal of the accessibility of health care and drug treatment services which should be provided to PWUD according to WHO criteria, i.e., among others, geographical accessibility, availability of low-threshold services, comprehensive assessment and treatment plans, evidence-based pharmacological and psychosocial interventions, and medically supervised withdrawal [5, 20–22]. In this article, we focus in detail on the following main objectives: (1) to describe the opioid-using population regarding their sociodemographic characteristics, their health status, and their patterns of drug use; (2) to investigate their utilization of health care services and drug treatment services; and (3) to describe the barriers that restrain PWUD from utilizing drug treatment services. Based on these results, we develop recommendations for improving the effectiveness and efficiency of health and social protection services for PWUD in Kazakhstan and Kyrgyzstan.

## Methods

In order to achieve the objectives of the study, a mixed methods approach including quantitative and qualitative components was used. In this article, we present results from one of the sub-studies, namely the quantitative survey of registered and non-registered PWUD.

### Sampling procedure, recruitment, and inclusion criteria

In order to reach the “hidden population” not in contact with the health care or drug treatment system, we decided to stratify our sample in order to examine two groups of PWUD: Group A consists of PWUD who are registered with a NC (i.e., they are currently in contact with the public health care or drug treatment system), and group B consists of PWUD not registered with narcology (i.e., they are not in contact with the public health care or drug treatment system).

Group A was generated via the following sampling procedure: The local UNODC research teams selected the recruitment sites. Registered PWUD were recruited consecutively as they appeared at their NC. The sampling frame for the registered drug-using population is the Narcological Register.

In the case of the non-registered PWUD, non-probability sampling methods appeared to be more suitable [23, 24]. For this study, an approach similar to snowball sampling was used. In this approach, “seeds” were set by researchers, and every seed was supposed to recruit up to three members of the non-registered PWUD (first wave). These recruited people were supposed to recruit up to three further participants (second wave). After the second wave, the recruitment process was stopped in order to avoid recruitment of PWUD who all belong to only one (or very few) social network(s). This would have borne the risk not to attain enough variance regarding the desired data and information needed to answer our research questions.

The required sample size of 300 PWUD per province (150 for each group) was determined with “EpiInfo,” a software application for calculating sample sizes provided by the Centers for Disease Control and Prevention [25].

In Kazakhstan, the local research teams recruited PWUD in four cities of the province East Kazakhstan oblast and in four cities of the province Karaganda oblast. In Kyrgyzstan, recruitment took place in three provinces: Bishkek city, Osh oblast (four cities), and Chuy oblast (three cities, four villages).

The sampling procedure in Kazakhstan started at the end of March 2013 and lasted until end of May 2013. In Kyrgyzstan, the first interviews were conducted by end of August 2013 and the last at the beginning of November 2013. The recruitment of PWUD for group A took place in the office of the local NC. At the starting point of the recruitment period, all registered PWUD who visited the NC were informed about the study, were asked for permission for a screening, and were screened consecutively. If a person met the inclusion criteria, the interviewee was informed about the voluntariness and anonymity of the participation and, if willing to participate, was then asked to sign the informed consent form and the interview was conducted.

Inclusion criteria are as follows:

- Currently registered with narcology and opioids as main drug, i.e., opioid use as reason for registration at NC → allocation to group A
- Currently not registered with narcology (self-reported) and current opioid use, i.e., at least once in the last 30 days (self-reported) → allocation to group B
- Not yet interviewed in this study
- Age 16 years or older
- Understanding of Russian or local language
- Cognitively able to follow the interview
- Not psychotic
- Willing to participate and having signed the informed consent sheet

The interviews took place partly in a separate room in the NC and partly in a scheduled appointment outside the NC, e.g., at trust points (low-threshold services that provide safe injecting equipment, information on safe drug use or safe sexual behavior, etc. [26]). The interviewers were given prior training by UNODC staff who provided instruction regarding filtering processes (i.e., questions about health services in prison should only be asked to interviewees with prison experience).

The members of group A were asked after their interview if they knew any non-registered PWUD. If they did so and if they were willing to recruit them for the study, they were given three contact cards which they were to hand out to a PWUD in their circle of acquaintances whom they regarded as potentially eligible for group B. The conduction of interviews with the members of group B was realized in other locations: some were interviewed in rooms of NGOs, some in AIDS centers or trust points, and some in rental apartments.

Screening and recruitment were conducted until the intended sample size of each group was reached. An incentive in the form of a pre-paid mobile phone card was given to all interviewees after the successfully conducted interview.

**Fig. 1:**
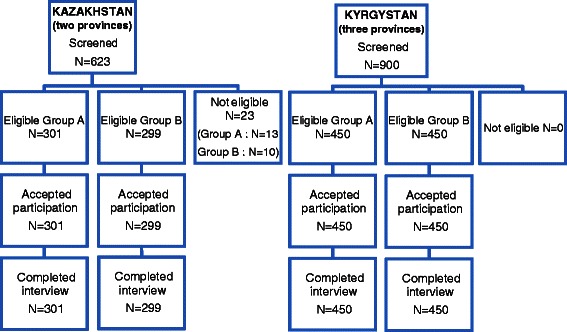
Scheme of sampling process

Figure 1 gives an overview of the sampling processes in Kazakhstan and in Kyrgyzstan.

### Data collection and processing

The data for the quantitative cross-sectional survey was collected by using a structured paper-based questionnaire containing approximately 100 questions. The questionnaire consisted of questions about the sociodemographic background of PWUD, their drug use patterns including risk behavior, questions about their physical and mental health status, PWUD’s accessibility and utilization of health services, and barriers to utilization of drug-related services. Some well-established measurement instruments were integrated into the questionnaire: the health assessment section from the Maudsley addiction profile [27] and questions from the treatment demand key indicator [28]. The time to conduct each interview averaged at about 50 min.

The UNODC data team entered the data and provided an Excel data set. Data were imported into SPSS software, and after running plausibility checks, data were analyzed using SPSS software for Windows, version 22. Differences between groups A and B were analyzed for significance using chi square tests and *t* tests, respectively.

## Results

### Sample characteristics

There were 600 participants recruited from Kazakhstan and 900 from Kyrgyzstan (see Table 1 for detailed sample characteristics). Participants in both groups, in both countries, were predominantly male. On average, registered PWUD were older than non-registered PWUD. Around two thirds of the interviewees specified their nationality as Russian. The only exception was group A from Kyrgyzstan where the proportion of Russians was merely half as big.

**Table 1:** Sample characteristics: sociodemographics, drug use behavior, health status, and overdose experience

	Kazakhstan		Kyrgyzstan	
Sociodemographics	Group A (reg. PWUD) (%)	Group B (non-reg. PWUD) (%)	Group A (reg. PWUD) (%)	Group B (non-reg. PWUD) (%)
	*N* = 301	*N* = 299	*N* = 450	*N* = 450
Male	76.4	75.3	87.8	71.6*
Age (years): mean (SD)	34.1 (7.8)	32.1 (8.3)*	39.5 (7.7)	36.3 (9.0)*
Nationality				
Russian	64.1	62.5	28.7	47.1*
Kazakh	22.9	21.1	0.7	0.9
Kyrgyz	1.0	2.0	18.2	18.0
Uzbek	0.7	1.0	24.4	16.4*
Others	17.2	14.7	28.0	19.4*
Stable housing^a^	86.2	86.9	89.9	74.9*
Stable partnership^b^	53.2	56.6	65.4	62.3
Living together with minor child/children^c^	58.0	60.5	72.5	80.7
Education: secondary school completed or higher	89.0	90.6	81.9	78.4
Employment situation				
Regularly employed^d^	52.1	54.8	37.2	38.5
Occasionally employed	29.1	28.6	39.1	53.5*
Drug use behavior				
Age at onset of regular use of heroin (years): mean (SD)	24.1 (7.0)	22.4* (7.9)	27.2 (7.6)	24.3* (5.7)
Age at onset of i.v. use (years): mean (SD)	20.7 (4.7)	21.0 (5.2)	25.2 (7.8)	22.8* (5.6)
i.v. use in the last 30 days^e^	99.0	97.1	98.6	92.8
12-month prevalence				
Heroin	93.2	94.5	50.8	94.4*
Opium	34.9	34.5	4.5	5.6
Cannabis	48.8	49.0	13.6	29.8*
Alcohol	76.3	83.6*	43.6	72.0*
30-day prevalence				
Heroin	68.8	70.2	15.3	83.8*
Opium	22.3	17.1	0.9	2.0
Cannabis	43.5	44.8	11.1	26.0*
Alcohol	71.4	77.9	39.6	69.1*
Health status and overdose experience				
Physical health score, mean (SD)^f^	14.2 (9.1)	11.9* (8.5)	11.5 (9.5)	9.4* (7.7)
Mental health score, mean (SD)^f^	11.7 (8.0)	10.3* (8.1)	9.9 (8.8)	8.5* (8.0)
HIV diagnosed (ever)	19.0	12.7	12.5	9.8
HCV diagnosed (ever)	56.3	30.4*	23.1	19.3
TB diagnosed (ever)	23.5	13.4*	13.3	20.1*
Opioid-related overdose (ever)	57.7	38.6*	48.7	68.9*

*PWUD* people who use drugs

*Indicates a significant result (*p* = 0.05 or smaller) between group A and group B in each country

^a^Stable accommodation includes living in own, parents’, or spouse’s apartment

^b^Stable partnership is based on the judgment of the respondent

^c^Database: only person with children

^d^Regularly employed includes regular, full-time, or part-time work as well as students

^e^Only people who consumed opioids in the last 30 days: KAZ: *N*_GroupA_ = 207, *N*_GroupB_ = 210; KRG: *N*_GroupA_ = 69, *N*_GroupB_ = 375

^f^Based on MAP: the sum score can range from 0 (very good) to 40 (very bad)

The social situation of most of the interviewees appeared to be relatively positive as the majority indicated their accommodation situation as stable; more than half of PWUD lived in a stable partnership (partnership stability was based on the judgment of the respondent). Further, around 60 % of both groups in Kazakhstan and up to 80 % in Kyrgyzstan lived together with minors (under 18 years old).

Most of the interviewees had completed at least secondary school education. This rather high level of education was also reflected in the employment rates: Most of the PWUD in this study were either regularly or occasionally employed.

In Kazakhstan, registered and non-registered PWUD only differed significantly in age; in Kyrgyzstan, this applied also to stable housing and occasional employment.

### Drug use behavior

In both countries, the mean age of onset for regular use of opioids was lower for non-registered PWUD compared to registered PWUD. This also applied to the age of first injecting drug use in Kyrgyzstan, but in Kazakhstan, there was no significant difference.

Intravenous drug use in the last 30 days was documented for almost all PWUD in both countries, and heroin was the dominant drug.

Regarding the 12-month prevalence of opioids, cannabis, and alcohol, the registered and non-registered PWUD in Kazakhstan did not differ significantly except for alcohol use. In Kyrgyzstan, however, the PWUD from group B had an explicitly higher drug consumption prevalence rate—except for opium—than the PWUD from group A. The same pattern was found in both countries regarding the 30-day prevalence.

### Health status

In both countries, non-registered PWUD had a better physical and mental health status than registered PWUD (see Table 1; lower scores correspond to better health status). The score values were comparable to those mentioned in the MAP user manual for the group of PWUD [27].

In general, all groups of PWUD in this sample showed high infection rates of diseases such as HIV (between 10 and 20 %), HCV (between 20 % and more than 50 %), and tuberculosis (TB) (between 13 % and almost 25 %). Furthermore, the prevalence of HIV as well as HCV infections was higher in group A than in group B without reaching significance in Kyrgyzstan. Regarding TB infections, the prevalence was higher in group B from Kyrgyzstan.

More than half (57.5 %) of the registered PWUD in Kazakhstan have ever experienced an opioid-related overdose. In Kyrgyzstan, two thirds of the non-registered PWUD have been affected by an overdose at least once in their lifetime, with this being a 20 % greater rate than among the registered PWUD.

### Access to health care

According to the World Health Organization (WHO), “preventing HIV transmission through injecting drug use is one of the key challenges to universal access in the health sector. A comprehensive package for the prevention, treatment and care of HIV among IDUs includes […] nine interventions.” [20, 22, 29]. The PWUD in our sample were asked about utilization of these nine interventions (see Table 2).

**Table 2:** Utilization of services

	Kazakhstan		Kyrgyzstan	
	Group A (reg. PWUD) (%)	Group B (non-reg. PWUD) (%)	Group A (reg. PWUD) (%)	Group B (non-reg. PWUD) (%)
	*N* = 301	*N* = 299	*N* = 450	*N* = 450
Utilization of nine interventions (ever)^a^				
HIV testing and counseling (T&C)	95.0	77.9*	97.1	62.4*
Prevention, diagnosis, and treatment of tuberculosis (TB)	93.0	81.9*	94.2	65.8*
Targeted information, education, and communication (IEC) for IDUs and their sexual partners	89.0	60.5*	95.3	63.8*
Needle and syringe programs (NSPs)	71.4	45.2*	76.0	46.9*
Opioid substitution therapy (OST) and other drug dependence treatment	80.7	15.7*	95.8	27.1*
Condom programs for IDUs and their sexual partners	69.8	41.8*	81.6	47.6*
Vaccination, diagnosis, and treatment of viral hepatitis	54.4	55.5	83.8	56.4*
Prevention and treatment of sexually transmitted infections (STIs)	53.3	52.5	82.7	55.3*
Antiretroviral therapy (ART)^b^	6.6	2.7*	9.6	6.2
None of the above	0.3	10.7*	0.2	24.2*
Utilization of drug-related services (ever)^a^				
Detoxification without methadone	46.3	9.2*	23.6	13.2*
Opioid substitution therapy	28.0	1.7*	90.4	0.4*
Psychotherapy	20.7	7.8*	11.3	8.1
Brief intervention/motivational interviewing/consulting	19.3	2.0*	11.6	8.7
Social rehabilitation programs	13.0	2.0*	3.8	4.5
Maintenance therapy with naltrexone or other opioid antagonist	11.3	5.1*	1.6	1.6
Detoxification with methadone	9.7	1.0*	20.4	4.3*
Relapse prevention training	7.0	1.0*	4.4	4.5
Self-help groups (12-step program)	5.3	1.4*	8.0	10.1
None of the above	19.0	84.0*	4.2	72.5*

*IDUs* injecting drug users, *PWUD* people who use drugs

*Indicates a significant result (*p* = 0.05 or smaller) between group A and group B in each country

^a^Multiple response

^b^Database: only HIV-infected PWUD

Except for antiretroviral therapy (ART), which was utilized only by very few PWUD (3–10 %), we could find high rates of utilization for all listed interventions. With the exception of vaccination/treatment of hepatitis in the Kazakhstan sample (which was not statistically significant), the registered PWUD showed higher rates of utilization than the non-registered drug users.^2^ Furthermore, one quarter of group B in the Kyrgyzstan sample as well as every tenth non-registered Kazakh PWUD declared never having utilized any services.

One of the most important drug treatment interventions regarding the improvement of health of injecting drug users is OST. With respect to specific interventions and drug treatments, the group differences became more apparent (see Table 2).

Detoxification without methadone and OST were the two main interventions as they showed the highest rates of utilization. OST was utilized by around one quarter of registered PWUD from Kazakhstan, whereas in Kyrgyzstan, more than 90 % reported having utilized OST. It is particularly worth mentioning that OST was hardly ever utilized by non-registered PWUD.

Furthermore, a large percentage (73–84 %) of the non-registered PWUD did not utilize any of the listed drug-related services at all. On the other hand, this applied also to almost a fifth of the registered PWUD from Kazakhstan.

In order to enhance the access of PWUD to the service providers, it is important to know why so many PWUD are not utilizing drug-related services. The barriers to utilization are shown in Table 3.

**Table 3:** Barriers to utilization of services

	Kazakhstan		Kyrgyzstan	
	Group A (reg. PWUD) (%)	Group B (non-reg. PWUD) (%)	Group A (reg. PWUD) (%)	Group B (non-reg. PWUD) (%)
	*N* = 55	*N* = 283	*N* = 8	*N* = 307
Barriers to utilization of drug-related services^a^				
I think I do not need treatment	76.4	60.9*	75.0	62.9
I think that current available treatment is not effective	29.1	29.0	25.0	16.3
I heard stories about the treatment regime that made me dislike it	25.5	19.3	62.5	9.8*
I heard stories about treatment staff that made me dislike it	16.4	15.1	50.0	5.2*
I cannot afford to pay for treatment/treatment is too expensive	14.5	15.5	50.0	17.3*
I do not trust governmental facilities	12.7	21.8	37.5	10.7*
I am afraid of problems with police if they know I was in treatment as a drug user	10.9	41.2*	37.5	19.9
Afraid that then everybody will know I am a drug user	9.1	41.2*	25.0	36.2
I do not want to be registered with narcology	7.3	51.3*	12.5	24.4

*PWUD* people who use drugs

*Indicates a significant result (*p* = 0.05 or smaller) between group A and group B in each country

^a^Multiple response, only people who never received drug-related treatment

The main reason was the belief that they do not require treatment. This was affirmed by the largest percentage of PWUD from both groups in both countries. Besides the fact that only very few registered PWUD (KAZ, *N* = 55; KRG, *N* = 8) answered this question at all, the results of the non-registered PWUD were of special interest: Interviewees from both countries indicated fear of registration with narcology, worries about problems with the police, and fear of becoming stigmatized by other people as important barriers to utilization. But also general mistrust of governmental facilities and skepticism of the effectiveness of the available treatment may have played a role in the decision of non-registered PWUD not to utilize drug-related treatment.

## Discussion

Illicit drug use, living conditions, and health status of PWUD, as well as their access and actual utilization of health care- and drug-related services were investigated for two groups that differ regarding their affinity to treatment providers and the official health care system. We assumed to find differences especially regarding access and utilization of services between PWUD being registered with a NC and non-registered PWUD.

The proportion of male persons among interviewed PWUD amounted to three quarters. The predominantly male gender of PWUD corresponded to international and regional findings [10, 30, 31]. In Kyrgyzstan’s group A, the proportion of males was noticeably higher. This could be explained by the greater proportion of men in OST which is the most frequently utilized drug-related service in this country.

Although the findings included well-known social problems such as homelessness or unemployment, it is of particular importance for the adjustment of the treatment system to tie in with positive findings such as stable accommodation (75 to 90 %), stable partnerships (53 to 65 %), and responsibility for minor children (60 to 80 %). This also means that drawing on existing familial resources is of great importance for social and health-related care.

In contrast to international research where high rates of unemployment of PWUD are often described, e.g., [10, 32], our study revealed a different picture: In Kazakhstan, where PWUD are generally younger, their employment situation was even more advantageous (more than 50 % in regular employment) than among Kyrgyzstan’s rather older PWUD (around one third in regular employment).

The results regarding drug use showed that both groups consisted of mainly long-term opioid-dependent persons who usually consumed intravenously. Further, we surveyed polyvalent consumption patterns with high proportions of (additional) cannabis and alcohol use.

Observing current consumption patterns, indications for an impact of the treatment system on these patterns were only found in group A from Kyrgyzstan. This can be explained by the broad implementation of OST in Kyrgyzstan. In this group, the consumption of heroin and all other substances was noticeably and significantly lower than in the group of non-registered PWUD.

In general, the health situation of PWUD was relatively poor, particularly regarding registered PWUD. One explanation for the worse health condition of registered PWUD might be their higher mean age.

HIV and HCV prevalence was higher in the whole sample if compared with existing epidemiological data from Kazakhstan and Kyrgyzstan [10, 13, 16]. In group A, this could be related to the fact that registered PWUD did have a poorer health in general. In group B, the high prevalence might be associated with the sampling procedure: The non-registered PWUD were often recruited in the periphery of institutions which cooperate with AIDS centers. Therefore, HIV- and/or HCV-infected PWUD who utilized services of these centers or trust points might have had a higher probability of being recruited for the study.

Regarding the results of the utilization of the “nine interventions” recommended by WHO [29], it was demonstrated that in both countries all interventions which showed significant group differences were more frequently utilized by PWUD from group A than from group B. This is particularly obvious regarding the intervention “OST and other drug dependence treatment.” In addition, we found high utilization rates of non-registered PWUD regarding general harm reduction measures, which indicates a high range of coverage of these measures. Though based on only a very small number of cases, the low utilization of ART (3 to 10 %) in the sample, however, suggests a need for action in improving the implementation of this important measure for HIV-infected PWUD.

Taking these results into account, group B seems to be an adequate sample to describe PWUD who are in contact with the low-threshold section of the health care system for PWUD, which is often provided in and around AIDS centers. The high rates of utilization suggest that the implementation of harm reduction measures for PWUD who are not integrated in the governmental health care system is successful. The respective situation in Kyrgyzstan seems to be even better than in Kazakhstan.

Regarding the utilization of OST and other drug dependence treatment, the difference between registered and non-registered PWUD is very clear; 84 % resp. 73 % of the non-registered PWUD in Kazakhstan and Kyrgyzstan declared no utilization at all, whereas these proportions were much lower in both groups of registered PWUD. In both countries, the most frequently utilized intervention in group B was detoxification without methadone. In Kazakhstan, OST—the most important intervention for opioid drug users—was utilized only by very few non-registered PWUD. Around 30 % of the registered Kazakh PWUD in our sample were treated with OST. As only very few PWUD in the whole country took part in OST programs [10], it is possible that our sample contains a bias in terms of an overrepresentation of OST clients.

Looking at the results from Kyrgyzstan, almost none of the non-registered PWUD reported ever having utilized OST which is an evidence-based and important treatment measure normally provided by NCs. This leads to the question which barriers prevent non-registered PWUD from utilizing treatment services. The most important barrier in all four groups was the assumption not to need treatment. This might indicate a lack of information about the severity of drug dependence as disease and the necessity of treatment. It is further conceivable that particularly those PWUD not (yet) living in social disintegration perceived no special need for treatment.

A further look at the barriers reported by non-registered PWUD in both countries reveals three main reasons. Firstly, these persons were either afraid of being registered or they mistrusted governmental facilities in general. Secondly, many non-registered PWUD thought that the provided treatment was not effective. Thirdly, they were afraid of being stigmatized or of getting in trouble if the police or neighbors and acquaintances found out that they were drug users. This concern (maybe based on bad experiences made by PWUD in the past) raises doubt regarding the extent of confidentiality on the side of the health care staff. The information deficit regarding the effectiveness of treatment as well as the mistrust of governmental facilities could be changed in a medium-term perspective; the reduction of stigmatization is a long-term task of society.

### Limitations

Besides the general problem of social desirability of answers to delicate questions (which are a particular issue for the PWUD in our study as they are often skeptical towards the public treatment system), the main limitation of the study is that the utilization of health services (“nine interventions”) is probably overestimated. This is related to the practical implementation of the sampling strategy in the selected regions. A bias may have occurred due to the access to group B: the recruiters were registered PWUD; therefore, the recruited members of the non-registered opioid users’ population have one thing in common: all of them know at least one person who is in the Narcological Register.

In addition, regarding recruitment problems, the access to both groups was realized in AIDS centers, trust points, and other locations where outpatient treatment is offered. These PWUD probably have a higher affinity to the health care system than other PWUD who could not be reached and could not be included in the sample. Therefore, the sampling strategy may have facilitated only a limited access to the so-called “hidden population” of PWUD.

## Conclusions

The general access to the health care system, especially for non-registered PWUD, has been opened through low-threshold facilities. However, utilization rates of non-registered PWUD show that the access to specific drug treatment is too limited and ought to be broadened. Besides that, the provision of OST should be expanded especially in Kazakhstan where the number of registered PWUD utilizing OST is rather low.

Furthermore, the access to OST should also be available for non-registered PWUD. This could be realized by involving existing trust points, as the fear of being registered with narcology (as a consequence of entering official drug treatment) was indicated by PWUD as one of the most important barriers regarding the utilization of official drug-related services. Trust points could further help overcome another important barrier of utilization: Many PWUD are convinced neither of their need for treatment nor of the effectiveness of drug treatment in general. By providing specific and target group-related information about modern drug treatment, these institutions can contribute to enhance the number of PWUD who utilize existing drug-related services. This could help individuals as well as the countries as a whole to deal with health and societal problems connected with drug use in a more effective way.

## Footnotes

1 In this document, the abbreviation PWUD is used to refer to opioid drug use. This is due to the fact that there is no official WHO abbreviation for this group of drug users. We did not choose the term “injecting drug user” (IDU) as most, but not all, PWUD in our sample are injecting drug users.

2 The health services for non-registered PWUD are provided mainly by AIDS centers (except for OST). Utilization of services in AIDS centers does not lead to a registration with the Narcological Register.
